# Acute toxicities of patients with locally advanced rectal cancer treated with intensified chemoradiotherapy within the CAO/ARO/AIO-12 trial: comparing conventional versus VMAT planning at a single center

**DOI:** 10.1038/s41598-022-25647-8

**Published:** 2022-12-08

**Authors:** Marcus Zimmermann, Anne Richter, Stefan Weick, Florian Exner, Frederick Mantel, Markus Diefenhardt, Emmanouil Fokas, Rebekka Kosmala, Michael Flentje, Bülent Polat

**Affiliations:** 1 Department of Radiation Oncology, University Hospital Würzburg, Josef-Schneider-Str. 11, 97080 Würzburg, Germany; 2 Department of Radiation Oncology, University Hospital Frankfurt, Frankfurt, Germany

**Keywords:** Rectal cancer, Radiotherapy

## Abstract

In locally advanced rectal cancer (LARC) neoadjuvant chemoradiotherapy is regarded as standard treatment. We assessed acute toxicities in patients receiving conventional 3D-conformal radiotherapy (3D-RT) and correlated them with dosimetric parameters after re-planning with volumetric modulated arc therapy (VMAT). Patients were randomized within the multicenter CAO/ARO/AIO-12 trial and received 50.4 Gy in 28 fractions and simultaneous chemotherapy with fluorouracil and oxaliplatin. Organs at risk (OAR) were contoured in a standardized approach. Acute toxicities and dose volume histogram parameters of 3D-RT plans were compared to retrospectively calculated VMAT plans. From 08/2015 to 01/2018, 35 patients with LARC were treated at one study center. Thirty-four patients were analyzed of whom 1 (3%) was UICC stage II and 33 (97%) patients were UICC stage III. Grade 3 acute toxicities occurred in 5 patients (15%). Patients with acute grade 1 cystitis (n = 9) had significantly higher D_mean_ values for bladder (29.4 Gy vs. 25.2 Gy, p < 0.01) compared to patients without bladder toxicities. Acute diarrhea was associated with small bowel volume (grade 2: 870.1 ccm vs. grade 0–1: 647.3 ccm; p < 0.01) and with the irradiated volumes V5 to V50. Using VMAT planning, we could reduce mean doses and irradiated volumes for all OAR: D_mean_ bladder (21.9 Gy vs. 26.3 Gy, p < 0.01), small bowel volumes V5–V45 (p < 0.01), D_mean_ anal sphincter (34.6 Gy vs. 35.6 Gy, p < 0.01) and D_mean_ femoral heads (right 11.4 Gy vs. 25.9 Gy, left 12.5 Gy vs. 26.6 Gy, p < 0.01). Acute small bowel and bladder toxicities were dose and volume dependent. Dose and volume sparing for all OAR could be achieved through VMAT planning and might result in less acute toxicities.

## Introduction

Neoadjuvant chemoradiotherapy (CRT) or short course radiotherapy (SCRT) followed by total mesorectal excision (TME) has been the standard treatment in locally advanced rectal cancer (LARC) of the middle and lower third^1^. Multimodal treatment sequence of neoadjuvant CRT or SCRT and TME led to improved locoregional control compared to adjuvant CRT or TME alone. Besides, preoperative CRT can increase sphincter preservation rates especially for tumors of the lower third^2–4^. However, long-term follow-up did not show a better disease-free survival (DFS) and overall survival (OS) foremost caused by distant recurrence^5,6^. Adjuvant chemotherapy after CRT and surgery failed to improve DFS or OS as well^7–9^. Recently, improvements in therapy outcome could be demonstrated in randomized phase II and phase III clinical trials following use of preoperative instead of postoperative chemotherapy referred to as total neoadjuvant treatment (TNT)^10–14^.

Radiation techniques for CRT include 3D-conformal radiotherapy (3D-RT) and intensity modulated radiation therapy (IMRT), such as volumetric modulated arc therapy (VMAT)^6,15,16^. Each of these is aiming to proper dose delivery to the target volume and sparing organs at risk (OAR). Radiotherapy and chemotherapy associated side effects might be detrimental for patients during treatment. Acute small bowel, genitourinary and sphincter toxicities have been evaluated in several studies^2,17–22^. Diarrhea and abdominal pain as symptoms of acute small bowel toxicity depend significantly on its irradiated volume^18,23^. Although acute genitourinary toxicities in radiotherapy are well examined for prostate cancer and seem to correlate with dose to the surface of the bladder, dosimetric evaluations for rectal cancer are lacking^24^. Quantitative data for acute and late anal sphincter toxicities are available for CRT and SCRT in LARC^19,20,25,26^. Nevertheless, analyses of correlation of dose parameters to sphincter function of pelvic radiotherapy are more focused on late than on acute adverse events. A dose and volume dependency has been found^22,27–29^. Therefore, reduction of radiation dose to OAR is important and might improve patient’s integrity during or immediately after treatment.

Several modern radiation and patient positioning techniques as well as patient preparations are taken into account for OAR sparing. Pelvic IMRT for CRT of LARC can reduce radiation doses to the urinary bladder, small bowel and anal sphincter^28,30,31^. This might improve clinical outcome for gastrointestinal and genitourinary toxicities^32,33^. Treatment in prone position with or without using a belly board is a common procedure to spare small bowel, a significant reduction of irradiated volumes has been shown^34,35^. Bladder filling protocols can decrease the dose to small bowel volume^36^.

This retrospective evaluation was performed to analyze acute toxicities of patients treated with 3D-RT as part of the TNT concept within the CAO/ARO/AIO-12 trial at the University of Würzburg^10^. Further we evaluated if VMAT planning might have resulted in less acute toxicities for these patients. Therefore, toxicities were evaluated during neoadjuvant CRT with 3D-RT and correlated with dosimetric parameters. The latter were compared to dose volume histogram (DVH) parameters of VMAT plans calculated retrospectively in a planning study. Furthermore, we analyzed whether patient conditions like small bowel anatomy, bladder filling or tumor localization can contribute to reduce dose to OAR.

## Methods

Patients with Union for International Cancer Control (UICC) 7th edition (2010) stage II and III rectal cancer treated within the multicenter, randomized phase II CAO/ARO/AIO-12 trial at our institution were included in this analysis.

All patients received TNT including long-term CRT with two concurrent cycles of continuous fluorouracil (250 mg/m^2^) on days 1–14 and days 22–35 and a 2-h infusion of oxaliplatin (50 mg/m^2^) on days 1, 8, 22 and 29. Three cycles of induction (ICT) or consolidation chemotherapy (CCT) consisting of a 2-h infusion of oxaliplatin (100 mg/m^2^), followed by a 2-h infusion of leucovorin (400 mg/m^2^), followed by a 46-h continuous infusion of fluorouracil (2400 mg/m^2^), repeated on day 15 were administered. Total radiation dose was 50.4 Gy in 28 fractions. Neoadjuvant treatment was followed by TME surgery. All patients gave written informed consent. The trial was approved by the ethics committee of the University of Frankfurt (Case No: 406/14) and was registered within ClinicalTrials.gov (NCT02363374, first posted 16/02/2015). All methods were performed in accordance with the declaration of Helsinki.

Patients had a planning computer tomography (CT) scan with a slice thickness of 3 mm with oral and rectal contrast in prone position using a belly board considered as a standard at our institution. Supine positioning was allowed if patients were not able to tolerate prone position. All patients were instructed to have a full bladder for CT-planning and each treatment fraction.

Contouring and treatment planning for 3D-RT or VMAT were performed using Pinnacle^3^ (Philips Radiation Oncology Systems, Fitchburg, WI). The primary tumor as well as suspect lymph nodes were defined as gross tumor volume (GTV). The clinical target volume (CTV) included the GTV, the mesorectum and the mesorectal, presacral and internal iliac lymph nodes. The inferior extent was at least 3 cm below the GTV. The superior extent was between vertebra level L5/S1. For patients with involvement of cranially located lymph nodes and tumors in the upper middle third of the rectum the cranial border was extended to vertebra level L4/L5. For the planning target volume (PTV) a margin of at least 5 mm was added to the CTV. Additional margins could be chosen by clinical judgement, for example in case of suspected lymph node metastases. Cranial and caudal field shrinkage was allowed after 25 fractions and was considered in DVH analysis. For this evaluation, we outlined OAR from 3 cm above the PTV to the anal verge in a standardized manner according to present literature for comparable dose evaluation^28,37^. Small bowel loops were individually contoured. The bladder outer surface was delineated from base to the dome. The structure corresponding to the bladder wall was created by contracting the bladder by 5 mm. The anal sphincter structure consisted of the internal and external sphincter muscles. Both femoral heads were delineated including the trochanter minor.

All patients were treated on a Elekta Synergy Agility linear accelerator (Elekta Oncology Systems, Crawley, UK).

Radiation dose was delivered using a 5-field technique 3D-RT with posterior and lateral field arrangement with additional segments. According to our internal guidelines for 3D-RT for rectal cancer, we choose beam angles of 90° (segment field), 100°, 260° and 270° (segment field) with a photon energy of 18 MV as well as 0° with a photon energy of 6 MV, respectively. Photon energies could be changed depending on the patient’s body type. PTV treatment dose was prescribed homogenous to the mean dose (within a range from 95 to 107%) (see Supplementary Figs. 1 and 2). Patient positioning and treatment preparation is described above. Image guidance was performed regularly according to our institutional standards using cone beam CT (CBCT). Special attention was paid on sufficient bladder filling.

For dose-evaluation we examined D_mean_ and D_max_ for bladder, bladder wall, small bowel, anal sphincter and femoral heads. Additionally, the small bowel, sphincter and femoral head volumes receiving doses between more than 5 to 50 Gy (V5-50) in 5 Gy steps were recorded. Absolute volumes for PTV, bladder, bladder wall, small bowel, femoral heads and sphincter were assessed.

During radiotherapy acute toxicities were assessed weekly classified according to Common Terminology Criteria for Adverse Events (CTCAE) version 4.03. Mean values of DVH parameters were correlated with associated toxicities. For each patient we retrospectively calculated a corresponding VMAT plan using the Pinnacle^3^ Auto-Planning Module and compared them with the clinically delivered 3D-RT plans. The standard for VMAT plans was 2 beams with rotations from 178° to 182° and 182° to 178°. The photon energy was 10 MV and the collimator angle was 15° for both arcs. The total dose of 50.4 Gy was prescribed to the mean dose (see Supplementary Fig. 1). For the PTV the D_98%_ should be more than 97% and the D_2%_ and D_max_ should be less than 106% and 107%, respectively.

For comparison of the different treatment plans and toxicities, t-test statistics were used to assess the relationship of dose and volume parameters if normal distribution was given. For not normally distributed parameters the Wilcoxon signed rank test and the Wilcoxon rank-sum test were performed. Normal distribution was assessed with the Kolmogorov–Smirnov test and the Shapiro–Wilk test. The chi-square test was used to compare toxicities caused by the treatment arms or the tumor localization in the middle or the lower third. For comparison of parameters with more than two categories we performed one factorial ANOVA. All statistical analyses were conducted using SPSS (SPSS Statistics, version 26, IBM Corporation, Armonk, New York, USA). A p-value < 0.05 was considered statistically significant.

## Results

### Patient characteristics

Between August 2015 and January 2018 35 patients were recruited at our center. Thirty-four patients were eligible for this analysis. One patient was withdrawn due to primary VMAT planning. Mean patient age was 61.6 years and 15 patients were male. Tumor stage was T3 in 100%, N0 in 3%, N1 in 15% and N2 in 82%. Eleven (32%) patients had a tumor in the lower, 23 (68%) patients in the middle third of the rectum. An upfront ileostomy was necessary in 2 (6%) cases. Seventeen (50%) patients were treated with ICT first and the other half received CCT. Patient, treatment and tumor characteristics are presented in Table 1.

**Table 1:** Patient, tumor, and treatment characteristics.

	Number (%), total n = 34
**Age (years)**	
Median (range)	61.6 (46.8–49.3)
**Sex**	
Male	15 (44)
Female	19 (56)
**ECOG score**	
0	31 (91)
1	2 (6)
2	1 (3)
**Clinical T category**	
cT3	34 (100)
**Clinical N category**	
cN0	1 (3)
cN1	5 (15)
cN2	28 (82)
**UICC stage**	
II	1 (3)
III	33 (97)
**CRM**	
Positive	23 (68)
Negative	11 (32)
**EMVI**	
Positive	16 (47)
Negative	17 (50)
Not known	1 (3)
**Histological grading**	
G1	4 (12)
G2	29(85)
Not known	1(3)
**Tumor location**	
Lower third	11 (32)
Middle third	23 (68)
**Upfront ileostomy**	
Yes	2 (6)
No	32 (94)
**Patient positioning**	
Prone	33 (97)
Supine	1 (3)
**Cranial PTV border**	
L5/S1	30 (88)
L4/5	4 (12)
**Treatment sequence**	
ICT + CRT	17 (50)
CRT + CCT	17 (50)

ECOG = Eastern Cooperative Oncology Group, UICC = Union for International Cancer Control, CRM = circumferential resection margin, EMVI = extramural venous invasion, PTV = planning target volume, L4 = lumbar vertebra 4, L5 = lumbar vertebra 5, S1 = sacral vertebra 1, ICT = induction chemotherapy, CRT = chemoradiotherapy, CCT = consolidation chemotherapy.

### Acute toxicities and correlation with DVH parameters

**Figure 1:**
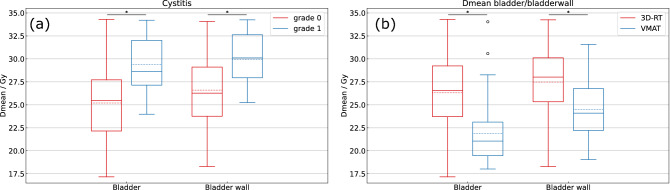
(**a**) Dependence of cystitis on Dmean bladder and bladder wall of all evaluated patients (n = 34) treated with 3D-conformal radiotherapy. Dose values are displayed as boxplots; box = interquartile range, solid horizontal line = median, dotted horizontal line = mean, whiskers = 1.5 × IQR. Statistical analysis was performed using t-test. A p-value < 0.05 was considered statistically significant. * = significant difference p < 0.01, Gy = Gray. (**b**) Reduction of Dmean bladder and bladder wall after volumetric modulated arc therapy (VMAT) re-planning compared to the 3D-conformal radiotherapy treatment plans. Dose values are displayed as boxplots; box = interquartile range, horizontal line = median, dotted horizontal line = mean, whiskers = 1.5 × IQR, dots = outliers. Statistical analysis was performed using Wilcoxon signed rank test. A p-value < 0.05 was considered statistically significant. * = significant difference p < 0.01, Gy = Gray, 3D-RT = 3D-conformal radiotherapy, VMAT = volumetric modulated arc therapy.

We could not see any differences with regard to bladder, small bowel or sphincter toxicities between both treatment sequences (p = 0.44, p = 1.0 and p = 0.65). During CRT acute grade 3 diarrhea and nausea were observed in 3 and 2 patients, respectively. Bladder and sphincter toxicities did not exceed grade 2. The maximum acute toxicities according to CTCAE are listed in Table 2. All dose and volume figures are stated as mean or median values. Acute cystitis did not exceed grade 1 and occurred in 9 patients (26%). Acute cystitis was not related to bladder (p = 0.51) or target volume (p = 0.92). There was a significant correlation with higher D_mean_ doses to the bladder (grade 0: 25.2 Gy versus (vs.) grade 1: 29.4 Gy; p < 0.01) and the bladder wall (grade 0: 26.6 Gy vs. grade 1: 29.9 Gy; p = 0.023) (Fig. 1a).

**Table 2:** Maximum acute bladder, small bowel and sphincter adverse effects during 3D-conformal chemoradiotherapy.

Toxicity	Number (%), total n = 34		
	Grade 1	Grade 2	Grade 3
**Small bowel**			
Diarrhea	11 (32)	14 (41)	3 (9)
Nausea	11(32)	7 (20)	2 (6)
Vomiting	6 (18)	2 (6)	0
**Bladder**			
Cystitis	9 (26)	0	0
Urinary urgency	18 (53)	1(3)	0
**Sphincter**			
Proctitis	18 (53)	9 (26)	0

Toxicities were classified according to Common Terminology Criteria for Adverse Events version 4.03.

**Figure 2:**
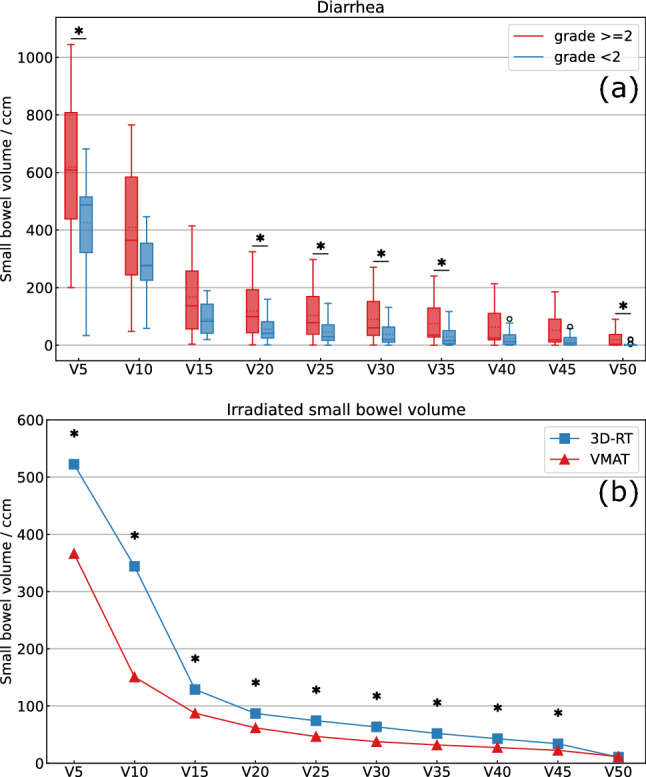
(**a**) Dependence of diarrhea on irradiated small bowel volume of all evaluated patients (n = 34) treated with 3D-conformal radiotherapy. Dose levels V5-50 are displayed as boxplots; box = interquartile range, solid horizontal line = median, dotted horizontal line = mean, whiskers 1.5 × IQR, dots = outliers. Statistical analysis was performed using Wilcoxon rank-sum test. A p-value < 0.05 was considered statistically significant. * = significant difference p < 0.05, ccm = cubic centimeter. (**b**) Reduction of irradiated small bowel volume receiving doses from 5 to 50 Gy after volumetric modulated arc therapy (VMAT) re-planning compared to the 3D-conformal radiotherapy treatment plans. Mean dose levels V5–V50 are displayed. Statistical analysis was performed using Wilcoxon signed rank test. A p-value < 0.05 was considered statistically significant. * = significant difference p < 0.01, ccm = cubic centimeter, 3D-RT = 3D-conformal radiotherapy, VMAT = volumetric modulated arc therapy.

Acute diarrhea was associated with the irradiated small bowel volume (grade < 2: 647.3 ccm vs. grade ≥ 2: 870.1 ccm, p < 0.01). This correlation was also significant for most dose levels from V5 to V50 (Fig. 2a). Neither the PTV nor the bladder volume had a significant impact on acute diarrhea (p = 0.78 and p = 0.45). Further, the D_mean_ to the small bowel did not correlate with diarrhea (p = 0.34). Acute proctitis did not exceed grade 2 and occurred in 26% of patients. The occurrence of proctitis did not depend on tumor localization (p = 1.0). However, the sphincter D_mean_ was significantly higher for tumors in the lower third (49.9 Gy vs. 28.8 Gy; p < 0.01). The extent of proctitis did not correlate with the D_mean_ to the sphincter (p = 0.54). This was also true for the subgroup of patients with tumors in the middle third (p = 0.26).

### Comparing 3D-RT treatment plans with retrospective calculated VMAT plans

The dose to OAR and the irradiated volume of OAR could be reduced using VMAT planning in comparison to conventional 3D planning. The D_mean_ to the bladder (21.9 Gy vs. 26.3 Gy, p < 0.01) and to the bladder wall (24.5 Gy vs. 27.5 Gy, p < 0.01) was significantly lower for VMAT plans (Fig. 1b). For the small bowel the D_mean_ (7.6 Gy vs. 11.3 Gy, p < 0.01), D_max_ (49.0 Gy vs. 50.1 Gy, p < 0.01) as well as the irradiated volumes V5–V45 (p < 0.01) were significantly reduced with VMAT planning compared to 3D-RT (Fig. 2b). Focusing on sphincter dose in all treated patients the D_mean_, V20 and V30 to V45 could be significantly reduced through VMAT planning. For the subgroups of patients with a tumor in the middle and the lower third, the sphincter D_mean_ could be reduced from 28.8 Gy to 27.5 Gy (p < 0.01) and from 49.9 Gy to 49.2 Gy (p = 0.02), respectively. Also for the femoral heads D_mean_, D_max_ as well as irradiated volumes V5 to V45 were significantly lower with VMAT planning than with 3D-RT (p < 0.01). Dose comparisons between VMAT and conventional 3D-RT plans are summarized in Table 3.

**Table 3:** Dose-volume parameters for 3D-RT and VMAT plans.

	Dmean (Gy)	Dmax (Gy)	V5 (ccm)	V10 (ccm)	V15 (ccm)	V20 (ccm)	V25 (ccm)	V30 (ccm)	V35 (ccm)	V40 (ccm)	V45 (ccm)	V50 (ccm)
**Bladder (mean volume 288.2 ccm)**												
3D-RT	26.3	51.0										
VMAT	21.9	51.7										
p-value	** < 0.01**	** < 0.01**										
**Bladder wall (mean volume 111.1 ccm)**												
3D-RT	27.5	50.1										
VMAT	24.5	51.7										
p-value	** < 0.01**	** < 0.01**										
**Small bowel (mean volume 759.0 ccm)**												
3D-RT	11.3	49.0	522.3	344.0	128.9	86.9	74.3	63.5	52.0	42.9	34.1	10.6
VMAT	7.6	50.1	366.3	150.7	87.4	61.8	46.8	37.7	31.9	27.4	22.6	11.5
p-value	** < 0.01**	** < 0.01**	** < 0.01**	** < 0.01**	** < 0.01**	** < 0.01**	** < 0.01**	** < 0.01**	** < 0.01**	** < 0.01**	** < 0.01**	0.57
**Sphincter (mean volume 15.5 ccm)**												
3D-RT	35.6	46.8	13.8	11.9	11.5	11.2	10.9	10.7	10.4	10.1	9.3	4.7
VMAT	34.6	46.3	13.6	11.9	11.3	11.1	10.7	10.3	10.0	9.4	8.8	4.8
p-value	** < 0.01**	0.10	0.13	0.86	0.10	**0.01**	0.09	** < 0.01**	** < 0.01**	** < 0.01**	** < 0.01**	0.83
**Femoral head right (mean volume 172.2 ccm)**												
3D-RT	25.9	46.6	140.1	129.4	123.3	119.6	110.7	93.6	68.9	18.2	1.0	0.1
VMAT	11.4	34.8	130.0	84.8	48.5	23.4	10.4	2.9	0.7	0.1	0.0	0.0
p-value	** < 0.01**	** < 0.01**	** < 0.01**	** < 0.01**	** < 0.01**	** < 0.01**	** < 0.01**	** < 0.01**	** < 0.01**	** < 0.01**	** < 0.01**	0.11
**Femoral head left (mean volume 170.5 ccm)**												
3D-RT	26.5	46.3	141.9	131.4	125.1	121.6	114.3	98.8	72.9	17.3	0.8	0.0
VMAT	12.5	36.5	132.2	89.6	56.9	29.1	11.1	4.0	1.2	0.2	0.0	0.0
p-value	** < 0.01**	** < 0.01**	**0.01**	** < 0.01**	** < 0.01**	** < 0.01**	** < 0.01**	** < 0.01**	** < 0.01**	** < 0.01**	** < 0.01**	0.32

Stated values are shown as mean values. Statistical analysis was performed using Wilcoxon signed rank test. A p-value < 0.05 was considered statistically significant. ccm = cubic centimeter, 3D-RT = 3D-conformal radiotherapy, VMAT = volumetric modulated arc therapy. Significant p-values are marked with bold.

## Discussion

We analyzed patients treated within the CAO/ARO/AIO-12 trial at one study center for acute adverse events and correlated them with DVH parameters. At the time of randomization treatment with 3D-RT was the internal standard technique for CRT of LARC. IMRT and VMAT techniques were preferred within the study protocol but 3D-RT with at least a three-field technique was also allowed. In our analysis acute grade 1/2 gastrointestinal toxicities were higher than in former trials using 3D-RT with concurrent 5-FU and Oxaliplatin. Genitourinary and sphincter toxicities as well as grade ≥ 3 gastrointestinal adverse events were in the same range or lower in our cohort^19,21^. We have to keep in mind that we assessed maximum acute toxicities during CRT and not at the end of neoadjuvant treatment as reported in the other trials. We therefore analyzed if there was any difference in acute toxicities caused by treatment sequence which we did not observe.

Oncologic outcome, treatment compliance, acute and late toxicities of the CAO/ARO/AIO-12 trial have been already published^10,14^. Compared to all eligible patients enrolled in the trial (n = 296), the acute toxicities at our center were higher for diarrhea (grade 1/2: 73% vs. 30%, grade 3/4: 9% vs.1%). This difference might be due to the applied 3D-RT treatment technique.

Thus, we re-calculated VMAT plans for each patient to perform DVH comparisons. We could hereby demonstrate dose sparing for all OAR. As the occurrence of acute cystitis was dependent on D_mean_ to the bladder and the bladder wall we would expect an improvement of tolerability for our patients. According to the literature DVH analyses of acute genitourinary toxicities after CRT of LARC are limited and are lacking for intensified chemotherapy regimens. Appelt et al. could show that doses to the bladder significantly correlated with cystitis in three dose–response models containing D_mean_, equivalent uniform dose or relative irradiated volume. Chemotherapy with tegafur uracil and treatment planning technique did not have an effect on dose response^38^. Otherwise, radiation effects on the bladder are well examined for gynecologic and urogenital cancers. However, radiation dose and irradiated subvolumes differ substantially and are not directly applicable to every tumor entity^39^^.^ For bladder cancer the whole or partial organ is recommended as target volume. Recommended total doses are ranging from 50 to 65 Gy and the disease itself might affect the toxicity. In cervical cancer treatment consists of a combination of external beam radiotherapy (EBRT) and a brachytherapy boost sometimes reaching cumulative local bladder doses up to 90 Gy (equivalent dose in 2 Gy fractions)^39^. Acute genitourinary toxicities of radiotherapy for prostate cancer seem to correlate with the dose to the bladder, whereby high doses are delivered to the inferior part and the trigone region, while sparing the upper parts^24,39,40^. Therefore, individual DVH analyses for current CRT treatments like TNT in LARC are needed. To further reduce bladder toxicity patients were advised to be treated with a full bladder to shift the anterior bladder parts outside of the radiation field. Besides, bladder distension is used to displace the small bowel from the target volume area^41^.

While the bladder volume had no impact on gastrointestinal acute toxicity, we could see significant higher grades of small bowel toxicity if larger small bowel volumes were located in the vicinity of the PTV. Moreover, a higher grade of diarrhea was found in relation to the irradiated volume over all dose levels V5 to V50. A significant reduction of the irradiated volume was achievable by using VMAT planning. In RCT for LARC a relationship of the irradiated small bowel volume and the degree of acute toxicity has been found and was quantified whereby the V15 was recognized as a threshold for higher grades^18,23^. In our analysis diarrhea grade ≥ 3 was low and occurred in only in 9% of patients. Therefore, we grouped patients with diarrhea to grade < 2 and grade ≥ 2 for statistical analysis. We feel that this threshold according CTCAE criteria already influences patient integrity during CRT. We could explain the low rate of toxicity grade 3 due to a mean V15 of 128.9 ccm. However, only nine patients (26%) exceeded a V15 of 150 ccm described as threshold by Baglan et al.^18^. None of them had diarrhea grade 3 and eight had diarrhea grade 2 corresponding to 57% of all patients with grade 2 small bowel toxicity. Due to VMAT re-planning the V15 could be decreased below 150 ccm for four of these nine patients and the mean V15 was reduced to 87.4 ccm. In retrospective trials a significant reduction of diarrhea grade ≥ 2 as well as grade 3 and 4 could be achieved by IMRT, respectively^32,33^. In our analysis, only acute gastrointestinal toxicities for 3D-RT were evaluable making a comparison with patients who were re-planned by VMAT impossible. However, comparing DVH parameters from our VMAT- planning study with the applied 3D-RT plans we assume a clinical benefit for the patients. VMAT planning was associated with a significant reduction of D_mean_, D_max_ and irradiated volumes V5 to V45, whereby the latter is known to correlate with small bowel toxicity as mentioned above.

This is different for acute sphincter toxicities. Proctitis is a common acute adverse event of CRT for LARC caused by exposition of the sphincter and the rectum to relevant radiation doses. We could not find any statistical difference for proctitis between the different tumor localizations, although we achieved dose sparing to the sphincter in tumors lying in the middle third. While the D_mean_ sphincter plays a role for sphincter late toxicity we could not find any correlation between the D_mean_ and acute proctitis^27^. This might be because proctitis is mainly caused due to radiation exposure of the rectum itself. Reviewing the literature, there are dosimetric evaluations of sphincter toxicities focusing on late adverse events especially on fecal incontinence. For LARC patients Arias et al. reported a significant but moderate correlation between dosimetric parameters and sphincter function. Fecal continence outcomes depended on distance of the tumor from the anal verge and patients with a V20 of 0 ccm had a lower incontinence score (measured with the Wexner-score)^27^. Within the CAO/ARO/AIO-04 trial we could also demonstrate an increase in the Wexner incontinence score from baseline 4 to 9 points at 5 years which was associated with a reduced quality of life^42^. In anal cancer anorectal dysfunction after definitive CRT was associated with D_mean_, D90 and V50 of the internal and external sphincter^43^. However extrapolating results from late toxicities to acute proctitis is not feasible. In our study, VMAT re-planning achieved a significant reduction of D_mean_ and irradiated volumes from V20 to V40. Despite that, there are no studies showing a reduction of acute proctitis by reducing the dose to the anal sphincter. For patients with tumors in the middle third dose and volume sparing with IMRT planning is achievable^28^. In their dose comparison Dapper et al. showed a significant reduction of D_mean_, D_median_, D_max_ and D_min_ as well as V10 to V40. This was also true for the D_mean_ and V20 to V40 in our analysis even though tumors in the lower third were included. Interestingly, the D_mean_ sphincter was significantly reduced for patients with tumors in the lower third what we did not expect because relevant parts of the sphincter were included in the PTV. The difference was only slight so that a clinical benefit remains uncertain. According to our data VMAT planning should be performed with regard to late sphincter toxicities even if acute adverse events could not be favorably affected.

We did not observe any acute complications with the hip bones during treatment. Femoral head and hip toxicities are known as late toxicities of pelvic radiotherapy^44^. Using VMAT planning we could significantly reduce dose and irradiated volumes of the femoral heads.

Limitations of this analysis are the small number of patients, different treatment sequences with ICT and CCT and no available toxicities for patients re-planned with VMAT. 3D-RT in prone position using a belly board and a bladder protocol was the internal standard treatment technique. Using intensified chemotherapy in a total neoadjuvant approach led us to reevaluate our toxicities. Strengths of this evaluation are that all patients were treated within a prospective randomized trial with pre-defined inclusion criteria and treatment schedule including target volume definitions. A balanced distribution to both treatment arms was shown in the main publication^10^.

Acute gastrointestinal and genitourinary toxicities of intensified CRT of LARC are dose and volume dependent. Radiation doses to the bladder, irradiated volumes of the small bowel, anal sphincter and femoral heads could be reduced with VMAT re-planning. This effort might reduce acute gastrointestinal and genitourinary toxicities when using upfront VMAT techniques. Acute proctitis was not associated with sphincter dose and tumor localization.

## Supplementary Information

Supplementary Information.

## Data Availability

All data analyzed during this study are included in this published article and its supplementary information files.
