# Supplementary material for: Acute toxicities of patients with locally advanced rectal cancer treated with intensified chemoradiotherapy within the CAO/ARO/AIO-12 trial: comparing conventional versus VMAT planning at a single center
Source: Sci Rep. 2022 Dec 8;12:21263. doi: 10.1038/s41598-022-25647-8 (PMC9731986; doi:10.1038/s41598-022-25647-8)
Supplement: Supplementary file 1 — Supplementary Information. [file 41598_2022_25647_MOESM1_ESM.pdf]

Acute toxicities of patients with locally advanced rectal cancer treated with intensified chemoradiotherapy within the CAO/ARO/AIO-12 trial: Comparing conventional versus VMAT planning at a single center

[1]Marcus Zimmermann, [1]Anne Richter, [1]Stefan Weick, [1]Florian Exner, [1]Frederick Mantel, [2]Markus Diefenhardt, [2]Emmanouil Fokas, [1]Rebekka Kosmala, [1]Michael Flentje, [1]Bülent Polat

Affiliations:

[1]Department of Radiation Oncology, University Hospital Würzburg, Würzburg, Germany

[2]Department of Radiation Oncology, University Hospital Frankfurt, Frankfurt, Germany

Corresponding Author:

Marcus Zimmermann, M.D.

Department of Radiation Oncology

University Hospital Würzburg

Josef-Schneider-Str. 11

97080 Würzburg

Germany

Tel.: +49 931 20128891

E-Mail: Zimmerman_m2@ukw.de

Supplementary Figures

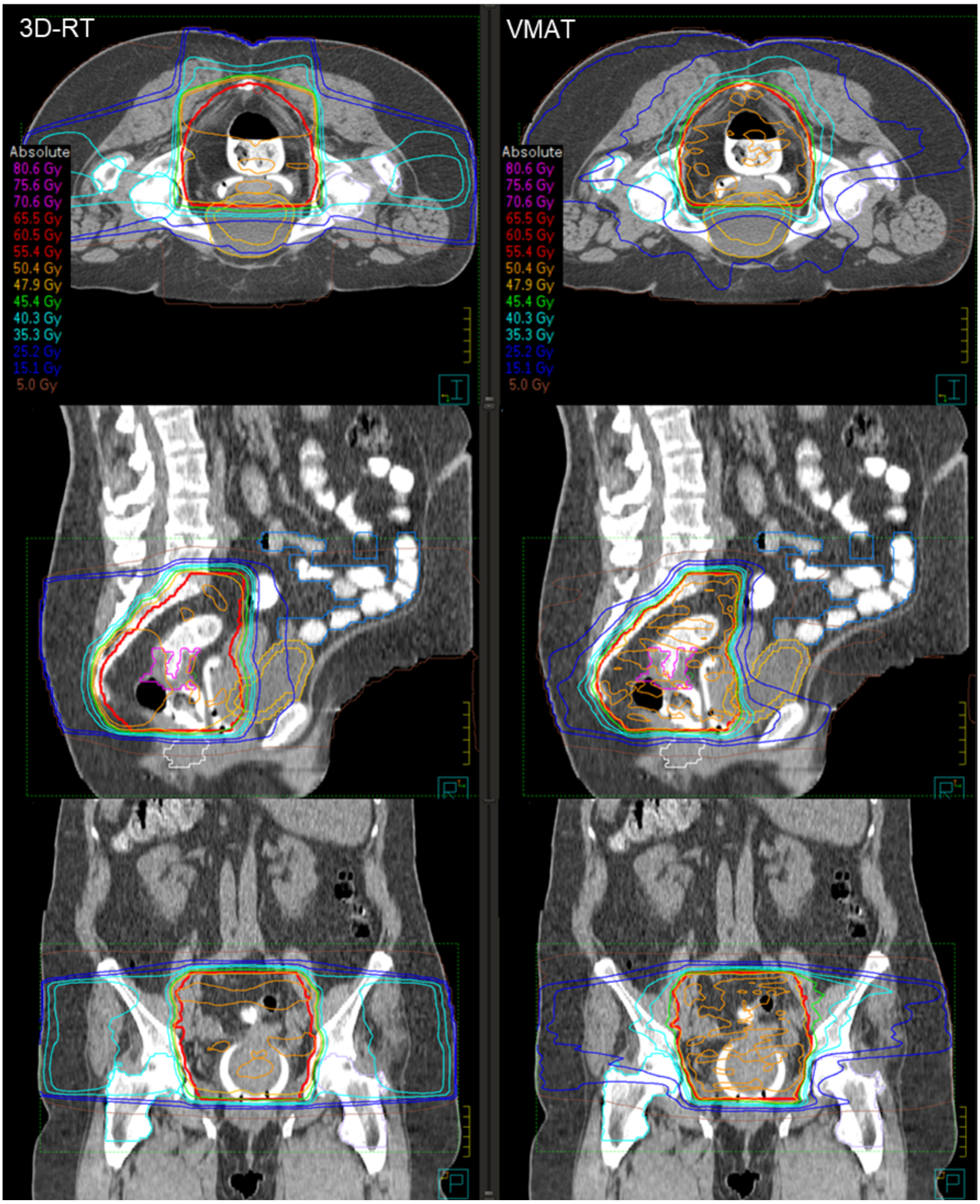

Supplementary Fig. 1: Dose distribution of a rectal cancer patient in axial, sagittal and coronal orientation. The 3D-conformal radiotherapy treatment plan is stated on the left side, the re-planned volumetric modulated arc plan is stated on the right side, respectively. Target volume and evaluated organs at risk are delineated: Red = planning target volume (PTV), purple = gross tumor volume (GTV), green = bladder, orange = bladder wall, blue = small bowel, white = anal sphincter, lavender = femoral head right, sky blue = femoral head left. Gy = Gray, 3D-RT = 3D-conformal radiotherapy, VMAT = volumetric modulated arc therapy.

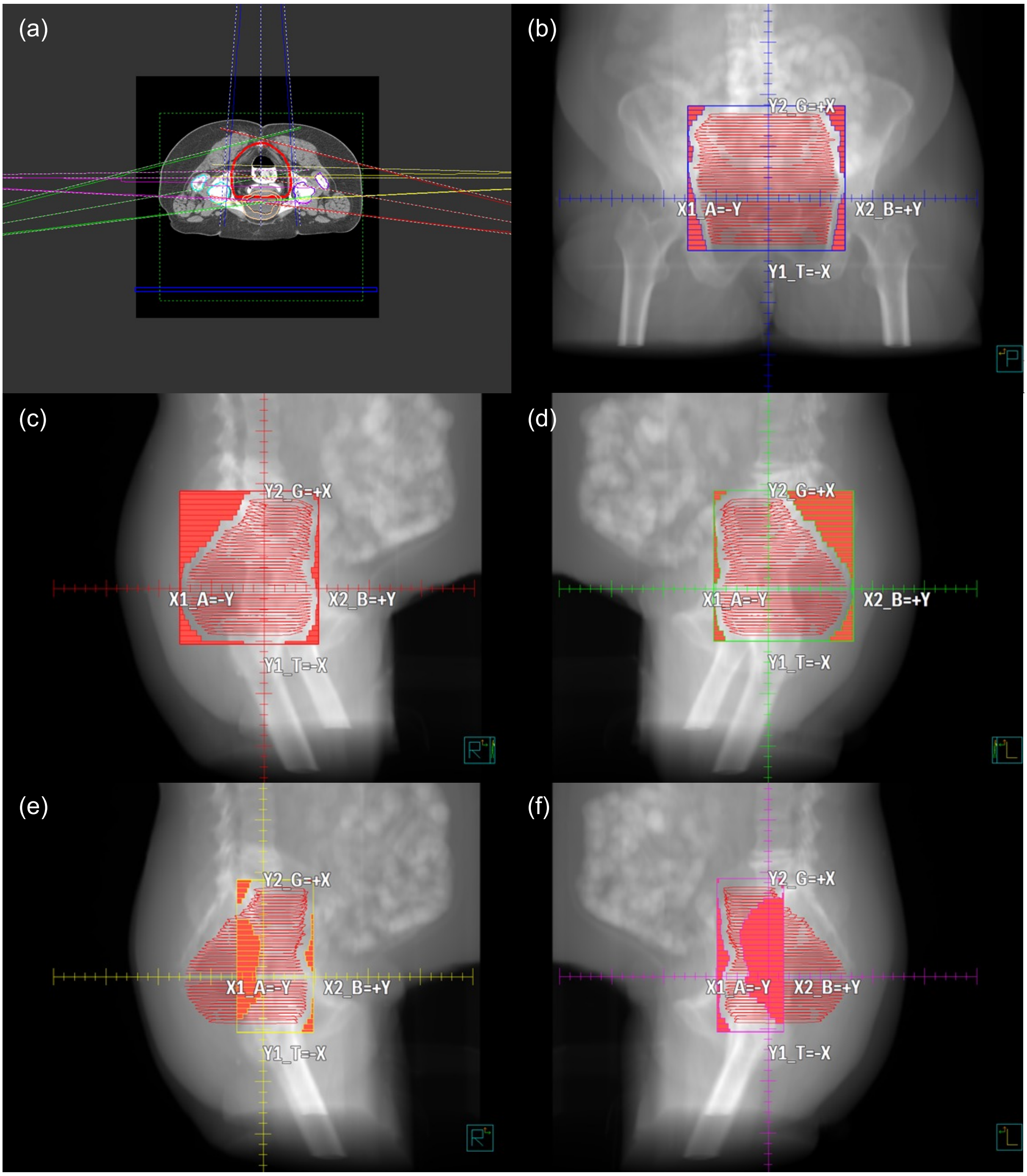

Supplementary Fig. 2: Field arrangement for a 3D-conformal radiotherapy treatment plan for a rectal cancer patient (same patient as shown in Supplementary Fig 1). Photon beams are illustrated in a planning computer tomography slice in axial orientation (a). Digitally reconstructed radiographs are stated for 0° (b), 100° (c) and 260° (d) fields as well as for 90° (e) and 270° (f) segment fields, respectively. The planning target volume (PTV) is delineated with red.

Supplementary raw data

#	Age	Sex	ECOG	T-Stage	N-Stage	M-Stage	UICC	CRM	EMVI
		1 = male						0 = CRM -	0 = EMVI -
		2 = female						1 = CRM +	1 = EMVI +
1	55.5	1	0	3	2	0	3	1	1
2	67.7	1	0	3	2	0	3	1	1
3	56.8	1	0	3	2	0	3	0	1
4	79.3	2	0	3	2	0	3	0	0
5	54.4	1	0	3	2	0	3	1	0
6	76.7	2	0	3	2	0	3	1	1
7	76.6	2	0	3	1	0	3	0	0
8	67.8	1	0	3	2	0	3	0	1
9	58.7	2	1	3	2	0	3	0	1
10	68.8	2	0	3	2	0	3	1	0
11	46.8	1	0	3	2	0	3	1	0
12	58.6	2	1	3	2	0	3	1	1
13	64.0	1	0	3	0	0	2	1	0
14	53.4	1	0	3	2	0	3	1	1
15	54.7	1	0	3	2	0	3	1	1
16	59.2	2	0	3	2	0	3	1	0
17	49.7	2	0	3	2	0	3	0	
18	65.1	2	0	3	1	0	3	0	1
19	64.8	2	0	3	1	0	3	1	0
20	51.0	2	0	3	2	0	3	1	1
21	51.2	2	0	3	2	0	3	1	0
22	57.7	2	0	3	2	0	3	0	0
23	55.5	2	0	3	2	0	3	1	0
24	68.1	2	0	3	1	0	3	1	0
25	51.6	1	0	3	1	0	3	1	0
26	65.0	2	0	3	2	0	3	0	0
27	67.1	1	2	3	2	0	3	1	1
28	62.7	1	0	3	2	0	3	1	0
29	66.1	1	0	3	2	0	3	1	1
30	58.6	1	0	3	2	0	3	1	1
31	60.5	2	0	3	2	0	3	0	0
32	72.1	2	0	3	2	0	3	0	0
33	71.5	1	0	3	2	0	3	1	1
34	72.8	2	0	3	2	0	3	1	1

#	Grading	Localization	Stoma	Positioning	cranial PTV border	Sequence
		1 = lower third	1 = yes	1 = prone	1 = L5/S1	1 = ICT + CRT
		2 = middle third	2 = no	2 = supine	2 = L4/L5	2 = CRT + CCT
1	2	1	0	1	2	2
2	2	2	0	1	1	1
3	2	2	0	1	1	2
4	2	2	0	1	1	1
5	2	1	0	1	1	2
6	2	2	0	1	1	2
7	2	2	1	2	1	2
8	2	2	0	1	1	2
9	2	2	0	1	1	1
10	2	2	0	1	1	1
11	2	1	0	1	1	2
12	2	1	1	1	1	1
13	2	2	0	1	1	1
14	2	2	0	1	2	1
15	1	2	0	1	2	2
16	2	1	0	1	1	1
17	2	2	0	1	1	2
18	2	2	0	1	1	2
19	2	1	0	1	1	1
20	2	2	0	1	1	2
21	2	1	0	1	1	1
22	2	2	0	1	1	2
23	2	2	0	1	1	1
24	2	2	0	1	1	1
25	1	1	0	1	1	2
26		2	0	1	1	2
27	2	2	0	1	1	1
28	1	1	0	1	1	1
29	2	1	0	1	2	1
30	2	2	0	1	1	1
31	2	2	0	1	1	2
32	1	2	0	1	1	1
33	2	1	0	1	1	2
34	2	2	0	1	1	2

#	Photon Energy 3D-RT [MV]	Arcs VMAT	Photon Energy VMAT [MV]
1	6 / 18	2	10
2	6 / 18	2	10
3	6 / 18	2	10
4	6 / 18	2	6
5	6 / 18	2	10
6	6 / 10	2	10
7	6 / 18	2	10
8	6 / 18	2	10
9	6 / 18	2	10
10	6 / 18	2	10
11	10	2	10
12	6 / 18	2	10
13	6 / 18	2	10
14	6 / 18	2	10
15	6 / 18	2	10
16	6 / 18	2	10
17	6 / 10	2	10
18	6 / 18	2	10
19	18	2	10
20	6 / 18	2	10
21	6 / 18	2	10
22	6 / 18	2	10
23	6 / 10	2	10
24	6 / 18	2	10
25	6 / 18	2	10
26	6 / 18	2	10
27	6 / 18	2	10
28	6 / 18	2	10
29	6 / 18	2	10
30	6 / 18	2	10
31	6 / 18	2	10
32	6 / 18	2	10
33	6 / 18	2	10
34	6 / 18	2	10

#	Acute gastrointestinal toxicity					Acute genitourinary toxicity	
	Diarrhea [°]	Diarrhea >=2°	Proktitis [°]	Nausea [°]	Vomiting [°]	Urge [°]	Cystitis [°]
		1 = yes, 0 = no					
1	0	0	1	0	0	0	0
2	2	1	1	1	0	1	1
3	1	0	0	1	1	1	0
4	1	0	0	0	0	1	0
5	0	0	0	1	0	1	0
6	2	1	1	1	0	2	0
7	1	0	0	0	0	1	1
8	3	1	2	0	0	0	0
9	1	0	1	2	1	1	0
10	3	1	2	1	1	1	0
11	0	0	2	1	0	1	1
12	1	0	0	0	0	1	0
13	2	1	1	0	0	0	0
14	2	1	2	1	0	1	1
15	2	1	1	1	0	0	0
16	2	1	1	2	1	0	0
17	2	1	1	3	1	0	0
18	2	1	2	2	2	0	0
19	2	1	1	2	0	0	0
20	0	0	0	0	0	0	0
21	2	1	2	3	1	0	0
22	1	0	1	0	0	0	0
23	1	0	2	2	2	1	1
24	3	1	2	2	0	0	1
25	0	0	1	1	0	1	0
26	2	1	1	0	0	1	0
27	1	0	0	1	0	1	1
28	1	0	2	0	0	0	0
29	1	0	1	0	0	1	0
30	2	1	1	0	0	1	1
31	0	0	1	1	0	1	0
32	1	0	1	2	0	0	0
33	2	1	1	0	0	0	0
34	2	1	1	0	0	1	1

#	PTV [ccm]
1	1218.13
2	1293.55
3	1175.21
4	1389.85
5	1502.79
6	1250.28
7	1299.34
8	1239.49
9	960.39
10	1368.15
11	1434.30
12	1357.50
13	1751.01
14	1544.20
15	2047.70
16	1239.12
17	1103.55
18	995.34
19	2408.81
20	1510.23
21	1506.70
22	1078.56
23	1045.20
24	1609.72
25	1364.93
26	1213.31
27	1835.98
28	1197.73
29	1618.11
30	1216.87
31	1165.79
32	1388.48
33	1196.02
34	1155.80

#	Bladder (BL) volume [ccm]	BL DMean [Gy]	BL Dmax [Gy]	BL DMean [Gy]	BL DMax [Gy]
		3D-RT	3D-RT	VMAT	VMAT
1	152.12	26.88	51.91	22.20	51.53
2	131.03	32.02	50.37	27.39	52.73
3	136.65	26.13	50.23	20.15	52.39
4	338.68	29.45	52.99	23.30	52.71
5	254.42	18.84	47.55	19.67	50.46
6	346.22	24.24	50.97	20.02	51.26
7	95.94	33.37	51.12	26.41	52.98
8	469.26	21.21	50.35	19.41	51.39
9	438.96	22.16	51.19	18.02	51.97
10	566.19	26.64	53.01	19.80	52.08
11	313.50	27.14	51.19	19.30	52.21
12	56.41	28.13	49.63	18.52	51.90
13	249.37	25.49	51.67	18.33	52.78
14	465.31	34.21	54.44	34.04	52.11
15	191.13	29.51	50.20	30.58	52.73
16	119.52	34.30	50.30	21.20	52.66
17	54.72	21.07	47.52	18.39	51.25
18	178.70	26.52	51.01	18.21	51.85
19	264.27	27.72	53.26	22.56	52.32
20	232.46	27.58	51.61	21.34	52.16
21	601.61	23.63	52.31	20.70	51.49
22	194.06	30.04	51.46	25.56	51.64
23	175.57	28.29	50.05	22.61	51.29
24	280.31	28.62	50.48	25.79	52.15
25	780.30	20.15	53.91	21.31	51.75
26	501.06	22.68	51.36	20.38	51.57
27	197.40	25.59	51.27	21.51	51.51
28	127.70	17.17	47.19	10.96	45.40
29	311.51	24.56	50.48	20.93	51.64
30	385.30	31.49	51.66	28.27	52.69
31	272.03	24.53	51.99	21.48	51.87
32	275.33	30.06	50.62	25.74	52.01
33	131.26	21.38	50.89	19.31	51.26
34	171.28	23.98	50.45	20.26	51.50

#	Bladderwall (BW) volume [ccm]	BW DMean [Gy]	BW Dmax [Gy]	BW DMean [Gy]	BW Dmax [Gy]
		3D-RT	3D-RT	VMAT	VMAT
1	84.00	27.48	50.91	24.95	51.53
2	75.09	32.63	50.37	30.45	52.73
3	75.53	28.10	50.23	23.73	52.39
4	124.44	31.31	52.99	27.67	52.71
5	105.55	19.96	47.54	21.99	50.46
6	130.64	26.27	51.08	23.01	51.26
7	54.38	33.04	51.14	28.07	52.98
8	149.79	23.45	50.17	22.06	51.39
9	150.90	23.76	51.21	21.14	51.97
10	181.93	29.27	53.06	24.96	52.08
11	118.93	27.95	51.25	22.71	52.21
12	42.41	29.01	49.58	20.48	51.90
13	116.12	25.59	51.61	20.03	51.96
14	178.38	34.25	54.44	36.02	52.11
15	85.70	29.11	50.07	31.57	52.47
16	76.36	34.06	20.28	23.44	52.66
17	43.34	22.53	47.52	19.04	51.25
18	99.36	28.19	51.01	21.88	51.85
19	110.99	28.20	53.26	23.92	52.32
20	105.45	30.12	51.61	24.69	52.16
21	194.11	26.04	52.31	25.18	51.49
22	89.55	30.34	51.46	27.30	51.61
23	98.74	28.41	50.05	23.36	51.92
24	109.55	30.11	50.48	28.06	52.15
25	227.12	22.78	53.79	24.12	51.75
26	161.82	25.09	51.36	24.79	51.57
27	88.41	26.63	51.27	24.50	51.51
28	65.50	18.30	47.19	12.96	45.40
29	121.95	25.93	50.48	24.41	51.64
30	135.96	31.10	51.66	29.31	52.69
31	112.18	26.23	51.99	24.07	51.87
32	112.74	31.36	50.62	28.99	52.01
33	69.86	22.44	50.89	21.13	51.26
34	79.24	25.26	50.45	22.90	51.50

#	Small bowel (SB) Volume [ccm]	SB DMean [Gy]	SB DMax [Gy]	SB V5 [ccm]	SB V10 [ccm]	SB V15 [ccm]
		3D-RT	3D-RT	3D-RT	3D-RT	3D-RT
1	521.64	18.45	52.03	406.01	344.13	189.30
2	365.85	10.68	51.27	225.05	144.58	55.09
3	825.34	12.66	51.47	681.59	445.92	147.75
4	1081.13	6.99	48.9	617.64	231.65	47.95
5	471.83	9.35	49.72	353.59	151.59	34.79
6	913.29	11.09	51.99	644.51	343.44	136.48
7	360.14	15.58	49.64	257.42	204.39	120.08
8	665.18	7.54	48.90	394.53	132.96	44.29
9	622.33	8.63	49.25	33.39	242.14	62.78
10	984.71	7.10	51.62	608.68	243.89	34.72
11	453.46	9.20	25.57	308.89	275.77	54.29
12	662.24	10.81	45.96	515.12	354.25	113.32
13	1156.01	13.85	51.26	883.93	583.64	257.45
14	663.04	9.38	51.65	399.65	205.17	72.24
15	1226.05	15.31	52.66	1038.89	660.21	332.88
16	824.99	23.97	52.07	807.81	764.80	414.34
17	573.04	16.19	50.38	438.56	364.31	184.66
18	1025.82	16.65	51.33	830.02	721.31	354.19
19	925.43	10.32	49.92	564.98	469.13	157.24
20	672.64	13.12	50.79	505.03	373.05	142.21
21	424.26	5.61	32.64	200.35	48.13	3.05
22	622.11	7.99	48.17	463.48	166.48	19.32
23	658.76	12.19	49.87	497.86	430.86	145.95
24	896.69	9.08	51.06	495.17	334.12	130.31
25	385.42	6.31	49.65	145.87	58.30	31.09
26	878.77	13.48	51.77	696.70	529.01	165.94
27	718.49	13.57	51.91	539.55	295.81	148.74
28	695.75	8.67	49.15	487.50	228.19	41.50
29	526.31	10.51	50.30	321.89	225.84	83.04
30	1357.06	15.13	52.02	1044.69	724.42	303.71
31	823.53	8.43	43.73	597.44	312.82	35.14
32	903.91	8.51	48.26	500.31	401.72	126.82
33	878.50	10.47	51.63	604.92	385.64	134.63
34	1032.75	7.57	49.08	647.26	299.97	56.80

#	SB V20 [ccm]	SB V25 [ccm]	SB V30 [ccm]	SB V35 [ccm]	SB V40 [ccm]	SB V45 [ccm]	SB V50 [ccm]
	3D-RT	3D-RT	3D-RT	3D-RT	3D-RT	3D-RT	3D-RT
1	159.58	144.92	131.05	116.98	90.90	57.95	20.51
2	43.25	38.02	33.69	28.20	21.36	16.06	2.78
3	107.60	91.84	72.60	50.34	35.83	26.17	4.47
4	38.38	28.83	19.36	8.89	4.17	1.65	0.00
5	24.81	20.84	16.43	11.54	8.33	6.46	0.00
6	101.09	84.97	73.67	62.03	54.95	46.70	15.94
7	81.20	70.41	62.83	55.06	47.02	32.03	0.00
8	30.92	23.65	17.10	7.88	2.43	0.43	0.00
9	41.56	31.41	21.89	16.66	13.13	7.61	0.00
10	15.23	10.36	6.63	3.28	1.36	0.57	0.13
11	0.91	0.01	0.00	0.00	0.00	0.00	0.00
12	44.33	26.95	16.35	7.21	2.91	0.03	0.00
13	192.47	168.62	151.46	129.02	110.16	92.20	47.48
14	58.24	50.25	41.92	30.65	18.53	10.73	1.27
15	257.39	226.11	190.54	152.07	121.31	90.14	43.01
16	324.79	297.39	270.69	239.89	213.46	185.33	8.75
17	132.94	114.82	100.02	85.10	72.28	57.60	3.23
18	220.39	188.60	168.97	146.10	126.42	107.20	54.95
19	99.61	78.11	59.78	35.11	20.84	11.67	0.00
20	88.84	78.52	68.37	58.49	52.51	42.57	3.24
21	1.05	0.41	0.10	0.00	0.00	0.00	0.00
22	10.57	7.20	4.98	3.20	1.79	0.48	0.00
23	59.22	43.93	36.22	29.76	22.58	14.58	0.00
24	67.2	50.66	40.58	31.39	23.17	13.7	1.27
25	22.96	16.74	10.28	4.64	3.15	2.02	0.00
26	121.12	104.89	91.02	74.59	63.88	55.10	36.87
27	127.17	115.00	101.55	88.08	76.67	64.12	18.84
28	24.72	21.00	18.39	15.30	12.19	7.53	0.00
29	62.37	53.32	44.05	30.05	18.27	12.25	0.82
30	250.34	232.25	217.00	200.49	187.11	174.33	89.57
31	14.36	9.39	5.40	1.35	0.11	0.00	0.00
32	28.31	15.86	9.93	4.30	2.49	0.91	0.00
33	79.20	62.17	44.65	33.52	24.86	18.79	7.06
34	23.36	17.41	12.02	7.14	3.75	1.40	0.00

#	SB DMean [Gy]	SB Dmax [Gy]	SB V5 [ccm]	SB V10 [ccm]	SB V15 [ccm]
	VMAT	VMAT	VMAT	VMAT	VMAT
1	12.81	52.76	402.39	256.34	156.93
2	5.64	54.65	133.32	37.58	20.13
3	6.77	51.81	398.96	105.69	51.20
4	5.75	49.9	555.56	84.68	30.66
5	5.25	51.28	171.87	32.33	17.38
6	6.28	53.03	241.80	94.44	69.64
7	10.52	51.89	213.33	89.72	57.91
8	4.77	47.84	222.50	44.11	24.57
9	5.65	51.80	241.78	58.78	29.51
10	3.91	49.43	201.72	37.59	16.08
11	3.38	31.05	77.85	6.34	2.10
12	5.60	48.65	282.44	51.65	24.60
13	8.84	52.90	640.64	241.05	135.65
14	7.07	51.53	288.79	179.31	136.66
15	13.76	52.31	1117.14	686.04	346.45
16	21.95	51.97	815.18	710.16	437.94
17	9.18	52.00	237.15	120.87	88.31
18	9.70	52.34	483.05	229.17	161.35
19	6.75	50.16	509.50	110.71	54.78
20	8.23	54.16	288.64	101.04	70.11
21	3.51	40.90	51.29	16.86	9.27
22	5.68	47.09	321.18	66.07	25.52
23	7.26	51.49	391.28	83.47	41.30
24	7.07	51.53	288.79	179.31	136.66
25	5.06	51.36	104.63	41.95	26.07
26	9.83	51.68	572.98	193.47	118.53
27	9.16	54.02	372.24	135.92	95.33
28	4.59	50.69	176.07	40.84	23.44
29	6.88	51.90	289.33	95.55	38.88
30	13.98	53.14	966.76	590.40	305.34
31	4.56	37.34	284.70	42.71	18.99
32	5.45	49.71	320.33	99.79	51.67
33	8.72	51.27	532.94	222.70	137.46
34	4.03	50.65	260.22	37.82	12.69

#	SB V20 [ccm]	SB V25 [ccm]	SB V30 [ccm]	SB V35 [ccm]	SB V40 [ccm]	SB V45 [ccm]	SB V50 [ccm]
	VMAT	VMAT	VMAT	VMAT	VMAT	VMAT	VMAT
1	92.81	58.37	38.89	27.96	20.29	13.62	6.03
2	14.59	11.58	9.28	7.40	5.70	3.98	2.14
3	37.62	30.56	25.65	21.60	18.12	14.74	5.91
4	21.28	15.21	10.51	6.63	2.54	1.11	0.00
5	13.00	10.19	8.26	6.79	5.69	4.33	0.56
6	59.03	53.03	49.05	45.99	43.66	41.04	15.64
7	48.14	41.51	36.02	31.02	26.22	20.76	11.01
8	14.54	8.82	4.86	1.94	0.58	0.06	0.00
9	21.09	15.65	12.95	10.08	8.10	6.35	2.35
10	10.04	6.44	3.93	2.07	0.91	0.26	0.00
11	1.07	0.55	0.04	0.00	0.00	0.00	0.00
12	14.77	9.03	5.54	3.10	1.34	0.20	0.00
13	106.21	90.39	78.65	68.53	59.90	49.08	27.01
14	93.26	54.04	30.71	20.26	15.84	11.96	7.49
15	196.99	131.64	96.96	74.62	58.78	46.31	27.85
16	300.89	228.93	194.83	173.80	154.96	134.63	62.07
17	72.70	61.44	52.29	44.79	38.63	30.86	13.86
18	128.61	111.64	98.28	86.36	74.46	60.44	25.84
19	39.96	31.04	23.95	17.73	11.20	3.36	0.01
20	60.98	55.47	49.96	46.06	41.05	34.27	13.93
21	5.07	1.96	0.86	0.28	0.02	0.00	0.00
22	9.95	2.47	1.04	0.60	0.26	0.01	0.00
23	31.47	25.76	20.58	17.49	14.72	10.00	0.89
24	93.26	54.04	30.71	20.26	15.84	11.96	7.49
25	16.11	8.45	4.50	3.10	2.01	1.62	0.62
26	94.57	80.68	68.78	60.07	53.32	46.96	30.67
27	81.92	73.39	66.32	59.58	51.79	39.23	6.18
28	16.08	12.82	11.22	9.55	7.91	5.70	0.68
29	26.45	19.09	14.46	10.71	7.10	4.03	1.14
30	233.55	206.59	188.93	178.21	168.17	160.05	118.10
31	9.79	3.84	0.78	0.05	0.00	0.00	0.00
32	30.56	17.24	8.72	4.56	2.78	0.79	0.00
33	95.90	53.21	29.58	23.36	18.41	11.76	2.68
34	8.25	4.93	3.05	1.73	0.91	0.37	0.01

#	Sphincter (SP) volume [ccm]	SP Dmean [Gy]	SP Dmax [Gy]	SP V5 [ccm]	SP V10 [ccm]	SP V15 [ccm]
		3D-RT	3D-RT	3D-RT	3D-RT	3D-RT
1	15.79	46.26	52.58	15.79	15.19	15.19
2	14.61	40.92	49.96	14.61	13.14	13.14
3	12.26	43.34	50.63	12.26	11.80	11.80
4	14.8	30.19	52.72	13.21	10.09	9.62
5	19.00	49.38	51.71	19.00	19.00	19.00
6	7.24	34.56	51.78	7.24	6.41	5.44
7	10.02	41.58	49.83	10.02	9.10	9.02
8	10.55	44.79	50.13	10.55	10.55	10.55
9	6.79	13.16	41.27	4.52	2.77	1.76
10	10.44	3.67	4.84	0.00	0.00	0.00
11	27.54	50.14	51.18	27.54	27.54	27.54
12	21.21	50.70	52.05	21.21	21.21	21.21
13	23.35	37.42	51.35	22.96	19.55	19.55
14	25.60	30.19	50.59	21.52	17.20	16.89
15	22.32	4.63	8.03	7.74	0.00	0.00
16	12.12	51.51	52.78	12.12	12.12	12.12
17	12.08	32.62	49.97	12.08	9.69	8.64
18	12.58	13.51	47.52	7.43	4.78	3.36
19	22.71	50.01	52.23	22.71	22.71	22.71
20	17.29	35.17	50.90	16.54	14.41	13.28
21	12.23	49.22	50.70	12.23	12.23	12.23
22	5.82	31.27	49.94	5.82	4.85	3.87
23	14.43	39.53	51.82	14.43	13.46	12.11
24	14.08	50.26	52.66	14.07	14.07	14.07
25	15.64	49.72	51.77	15.64	15.64	15.64
26	15.43	19.03	49.70	12.85	7.19	7.19
27	22.40	33.70	50.82	21.33	16.92	15.84
28	14.93	50.12	51.83	14.93	14.93	14.93
29	11.80	50.70	51.69	11.80	11.80	11.80
30	19.87	29.38	50.99	17.84	13.68	12.86
31	14.05	5.61	8.95	7.28	0.00	0.00
32	11.37	36.34	51.07	11.37	9.99	8.81
33	16.33	50.95	52.01	16.33	16.33	16.33
34	20.48	12.06	44.10	13.84	6.26	3.85

#	SP V20 [ccm]	SP V25 [ccm]	SP V30 [ccm]	SP V35 [ccm]	SP V40 [ccm]	SP V45 [ccm]	SP V50 [ccm]
	3D-RT	3D-RT	3D-RT	3D-RT	3D-RT	3D-RT	3D-RT
1	14.5	14.45	14.45	14.32	13.38	13.38	9.72
2	12.35	12.35	12.35	11.58	11.18	9.71	0.00
3	11.05	11.02	11.02	11.02	9.74	8.66	0.17
4	9.5	8.2	8.2	8.06	6.68	5.83	3.05
5	19.00	19.00	19.00	18.81	18.25	18.18	10.33
6	5.44	5.40	4.09	4.09	4.09	3.09	1.27
7	8.67	8.59	8.59	8.40	7.57	6.52	0.00
8	10.37	9.82	9.82	8.96	8.95	8.01	0.02
9	1.76	1.03	0.81	0.81	0.63	0.00	0.00
10	0.00	0.00	0.00	0.00	0.00	0.00	0.00
11	27.54	27.54	27.54	27.54	27.54	27.50	20.73
12	21.21	21.21	21.21	21.21	21.21	21.21	19.16
13	17.32	17.32	17.32	15.35	15.35	13.11	5.19
14	15.47	14.94	14.94	14.60	12.85	10.75	0.08
15	0.00	0.00	0.00	0.00	0.00	0.00	0.00
16	12.12	12.12	12.12	12.12	12.12	12.12	11.87
17	8.64	7.91	7.39	7.39	6.44	5.21	0.00
18	3.36	2.10	2.10	2.10	1.11	1.11	0.00
19	22.71	22.71	22.71	22.71	22.71	22.71	9.35
20	12.12	12.12	11.15	10.85	10.65	9.03	2.14
21	12.23	12.23	12.23	12.23	12.23	11.31	2.93
22	3.87	3.87	3.69	2.74	2.74	1.40	0.00
23	12.11	11.36	10.57	10.57	10.46	8.98	3.89
24	14.07	14.07	14.07	14.07	14.07	14.07	8.77
25	15.64	15.64	15.64	15.64	15.64	15.58	6.72
26	5.03	5.03	5.03	3.21	3.21	1.75	0.00
27	15.34	15.34	14.25	13.64	13.64	11.49	1.86
28	14.93	14.93	14.93	14.93	14.93	14.93	9.96
29	11.80	11.80	11.80	11.80	11.80	11.80	10.58
30	12.86	10.70	10.70	10.70	8.15	7.55	2.74
31	0.00	0.00	0.00	0.00	0.00	0.00	0.00
32	8.81	7.52	7.52	7.52	7.06	6.18	2.53
33	16.33	16.33	16.33	16.33	16.33	16.33	15.84
34	3.85	3.85	1.93	1.93	1.93	0.00	0.00

#	SP Dmean [Gy]	SP Dmax [Gy]	SP V5 [ccm]	SP V10 [ccm]	SP V15 [ccm]
	VMAT	VMAT	VMAT	VMAT	VMAT
1	44.21	51.01	15.79	15.58	15.19
2	39.97	51.71	14.61	13.14	12.35
3	42.04	51.27	12.26	11.80	11.53
4	29.79	51.38	14.8	11.57	9.63
5	48.40	51.66	19.00	19.00	19.00
6	28.31	49.53	7.24	5.68	4.86
7	38.11	50.82	10.02	9.02	8.59
8	43.78	50.99	10.55	10.55	10.02
9	12.23	35.89	4.52	2.77	1.76
10	3.70	4.81	0.00	0.00	0.00
11	49.61	52.38	27.54	27.54	27.54
12	50.43	52.26	21.21	21.21	21.21
13	36.13	51.15	22.96	19.56	18.65
14	27.73	51.28	20.23	16.89	15.10
15	4.11	7.66	5.16	0.00	0.00
16	50.67	51.71	12.12	12.12	12.12
17	28.36	49.25	11.31	8.64	8.48
18	13.86	46.51	7.43	4.78	3.36
19	49.95	52.10	22.71	22.71	22.71
20	33.91	51.80	16.22	13.40	12.60
21	49.65	51.17	12.23	12.23	12.23
22	28.81	47.24	5.82	4.85	4.34
23	39.61	50.94	14.43	13.56	12.92
24	49.50	51.21	14.07	14.07	14.07
25	49.76	51.66	15.64	15.64	15.64
26	16.90	47.04	11.25	7.19	5.62
27	31.42	50.90	20.02	16.02	15.34
28	48.67	51.38	14.93	14.93	14.93
29	50.29	51.31	11.80	11.80	11.80
30	31.48	49.96	17.84	14.56	14.56
31	6.13	12.19	7.28	2.01	0.00
32	35.99	50.25	11.37	9.99	8.96
33	50.35	51.25	16.33	16.33	16.33
34	11.18	41.03	13.84	6.26	3.88

#	SP V20 [ccm]	SP V25 [ccm]	SP V30 [ccm]	SP V35 [ccm]	SP V40 [ccm]	SP V45 [ccm]	SP V50 [ccm]
	VMAT	VMAT	VMAT	VMAT	VMAT	VMAT	VMAT
1	14.45	14.45	13.42	13.38	12.83	11.92	4.38
2	12.35	11.85	11.18	10.84	10.10	9.19	4.94
3	11.02	11.02	9.99	9.74	9.72	8.23	1.54
4	8.59	8.2	8.01	6.87	6.66	5.23	2.02
5	19.00	18.25	18.25	18.22	17.40	17.13	12.72
6	4.09	4.09	3.22	3.09	2.98	2.21	0.00
7	8.56	7.58	7.57	6.70	6.23	5.06	1.64
8	9.82	9.61	8.95	8.95	8.02	7.68	2.82
9	1.76	0.81	0.81	0.14	0.00	0.00	0.00
10	0.00	0.00	0.00	0.00	0.00	0.00	0.00
11	27.54	27.54	27.54	26.88	26.53	25.67	20.70
12	21.21	21.21	21.21	21.21	21.21	20.74	18.15
13	17.32	16.89	15.60	15.35	14.70	12.63	3.77
14	14.94	13.23	12.85	12.16	10.57	8.42	2.21
15	0.00	0.00	0.00	0.00	0.00	0.00	0.00
16	12.12	12.12	12.12	12.12	12.12	12.12	11.93
17	7.39	7.39	5.77	5.77	4.10	3.74	0.00
18	3.36	2.18	2.10	2.08	1.11	1.07	0.00
19	22.71	22.71	22.71	22.71	22.71	22.71	10.85
20	12.12	11.98	10.85	10.85	9.17	8.38	3.86
21	12.23	12.23	12.23	12.23	12.23	11.31	8.71
22	3.87	3.66	2.74	2.74	1.46	1.40	0.00
23	12.11	11.95	10.57	10.57	9.13	8.91	2.24
24	14.07	14.07	14.07	14.07	14.07	14.07	6.83
25	15.64	15.64	15.64	15.64	15.20	15.16	10.95
26	5.03	5.03	3.21	3.21	1.43	1.40	0.00
27	13.95	13.64	13.26	12.00	11.85	10.17	1.22
28	14.93	14.93	14.93	14.53	13.89	13.02	8.84
29	11.80	11.80	11.80	11.80	11.80	11.80	9.58
30	12.86	12.86	12.37	10.70	10.27	7.95	0.00
31	0.00	0.00	0.00	0.00	0.00	0.00	0.00
32	8.81	8.04	7.52	7.52	6.18	5.78	0.50
33	16.33	16.33	16.33	16.33	16.33	16.33	14.27
34	3.85	2.68	1.93	1.83	0.53	0.00	0.00

#	Femoral head_L (FHL) volume [ccm]	FHL Dmean [Gy]	FHL Dmax [Gy]	FHL V5 [ccm]	FHL V10 [ccm]	FHL V15 [ccm]
		3D-RT	3D-RT	3D-RT	3D-RT	3D-RT
1	135.73	27.24	49.18	117.37	110.12	104.97
2	179.35	24.99	42.17	151.81	140.74	134.44
3	163.59	26.86	44.07	132.39	120.59	108.85
4	155.51	29.84	49.54	145.37	136.53	130.82
5	199.21	30.17	41.68	189.12	184.51	181.30
6	196.05	23.24	44.26	156.57	139.53	131.35
7	135.30	30.82	47.86	126.66	120.90	118.59
8	188.10	31.09	45.19	176.56	163.44	151.76
9	152.71	11.53	49.52	67.01	53.40	46.22
10	144.91	19.71	51.02	101.91	87.64	81.51
11	253.56	27.52	44.13	223.14	198.90	191.00
12	156.97	25.61	48.34	133.75	122.74	119.71
13	254.76	27.56	46.88	211.81	193.78	176.88
14	208.74	29.81	42.59	186.77	171.87	156.25
15	266.30	23.27	42.98	190.73	173.61	166.98
16	128.12	29.06	47.07	110.55	102.54	97.03
17	136.84	22.04	47.64	98.99	88.25	80.81
18	131.58	18.82	48.90	81.21	72.66	66.23
19	128.23	32.69	49.93	125.86	121.66	120.18
20	158.27	22.69	47.73	138.51	124.62	116.30
21	160.74	26.36	42.70	127.30	119.92	118.27
22	108.50	36.40	44.60	108.09	105.35	104.02
23	92.12	23.92	47.82	72.13	66.75	61.97
24	164.82	34.19	48.57	164.82	164.80	161.96
25	169.72	33.94	48.60	164.79	155.02	151.29
26	149.41	11.71	41.17	73.31	59.10	51.82
27	221.69	28.17	49.45	192.23	181.32	177.62
28	216.37	31.43	43.36	199.90	191.37	188.61
29	243.78	32.89	47.73	221.50	208.77	200.77
30	202.47	15.76	41.72	117.35	99.97	90.38
31	140.53	24.39	48.34	110.10	101.23	92.09
32	143.89	27.01	48.71	124.61	115.28	109.76
33	183.37	35.11	42.63	183.37	181.03	178.97
34	126.10	24.96	48.15	98.62	91.20	85.03

#	FHL V20 [ccm]	FHL V25 [ccm]	FHL V30 [ccm]	FHL V35 [ccm]	FHL V40 [ccm]	FHL V45 [ccm]	FHL V50 [ccm]
	3D-RT	3D-RT	3D-RT	3D-RT	3D-RT	3D-RT	3D-RT
1	101.61	94.23	79.81	58.58	6.69	3.27	0.00
2	128.52	112.56	86.31	51.23	1.67	0.00	0.00
3	105.98	104.24	101.71	92.08	25.22	0.00	0.00
4	128.60	118.88	104.97	73.56	11.87	1.57	0.00
5	176.94	155.48	123.50	83.00	5.57	0.00	0.00
6	125.64	101.26	84.11	57.77	15.14	0.00	0.00
7	116.32	110.59	87.28	63.74	19.24	0.27	0.00
8	148.80	146.12	132.01	111.92	40.36	0.01	0.00
9	40.45	34.00	24.95	18.76	4.05	1.05	0.00
10	78.31	71.60	50.89	26.88	4.40	1.31	0.05
11	187.67	182.99	165.30	102.79	16.19	0.00	0.00
12	116.83	102.22	72.78	45.78	10.52	0.22	0.00
13	172.51	169.28	165.16	151.42	33.38	0.06	0.00
14	152.13	150.03	147.61	142.15	32.28	0.00	0.00
15	163.09	156.27	134.55	109.12	11.30	0.00	0.00
16	95.01	93.67	87.01	72.48	15.24	0.03	0.00
17	77.76	74.74	61.28	48.04	6.52	0.77	0.00
18	63.77	59.15	53.30	37.75	2.32	0.62	0.00
19	118.60	115.41	107.42	59.01	8.46	4.59	0.00
20	106.00	74.89	45.16	26.55	3.16	0.12	0.00
21	114.07	111.28	88.60	69.13	14.65	0.00	0.00
22	102.99	101.69	94.10	84.40	33.70	0.00	0.00
23	60.46	56.56	48.04	25.35	2.34	0.77	0.00
24	160.46	157.81	146.84	81.06	4.45	1.82	0.00
25	149.76	144.31	130.14	113.81	56.95	0.75	0.00
26	45.03	32.25	17.14	7.64	0.16	0.00	0.00
27	174.95	165.14	131.80	91.34	20.30	2.87	0.00
28	186.82	183.50	162.16	119.33	20.82	0.00	0.00
29	197.79	195.00	185.88	166.03	99.51	0.46	0.00
30	83.63	71.91	52.83	33.71	1.90	0.00	0.00
31	87.68	86.16	71.48	52.54	9.76	1.94	0.00
32	107.08	102.15	91.33	32.34	9.51	5.21	0.00
33	178.04	173.90	151.29	113.90	33.81	0.00	0.00
34	82.45	78.48	71.22	56.88	5.07	0.77	0.00

#	FHL Dmean [Gy]	FHL Dmax [Gy]	FHL V5 [ccm]	FHL V10 [ccm]	FHL V15 [ccm]
	VMAT	VMAT	VMAT	VMAT	VMAT
1	14.10	43.71	112.18	95.68	50.74
2	12.28	36.32	154.61	99.57	58.40
3	9.05	35.75	93.83	50.22	33.98
4	16.40	44.56	137.74	104.79	74.00
5	11.37	23.65	196.40	119.09	32.52
6	11.95	35.08	153.89	126.37	68.24
7	19.23	40.12	131.71	119.25	103.41
8	13.40	37.50	168.55	119.65	76.54
9	6.77	34.37	63.01	37.85	23.55
10	14.88	41.77	124.51	108.56	72.91
11	7.91	30.10	178.68	51.41	21.90
12	15.97	38.89	138.34	118.62	93.46
13	15.13	41.76	209.29	178.50	145.52
14	6.13	20.59	130.00	23.61	2.31
15	4.40	23.95	79.42	21.71	6.00
16	15.84	40.86	109.45	95.46	82.43
17	11.94	30.88	94.22	78.83	57.16
18	10.53	45.43	65.94	45.48	34.95
19	17.18	49.01	128.23	112.75	73.00
20	16.68	35.07	153.59	134.57	93.33
21	7.90	28.65	114.19	46.62	14.32
22	18.67	34.69	105.35	101.38	88.71
23	15.29	43.83	70.64	63.83	47.83
24	15.23	47.89	162.15	88.75	60.52
25	11.95	35.04	151.02	99.90	57.37
26	5.67	31.41	59.12	25.97	13.33
27	13.20	41.91	192.40	141.53	70.72
28	11.70	31.15	190.78	119.24	66.04
29	12.31	33.00	230.28	136.25	71.09
30	7.90	35.90	110.45	64.33	37.22
31	12.23	35.40	106.53	86.33	50.79
32	16.14	44.52	128.01	111.39	80.00
33	8.33	29.90	157.65	41.74	12.50
34	14.59	39.78	92.13	78.29	59.32

#	FHL V20 [ccm]	FHL V25 [ccm]	FHL V30 [ccm]	FHL V35 [ccm]	FHL V40 [ccm]	FHL V45 [ccm]	FHL V50 [ccm]
	VMAT	VMAT	VMAT	VMAT	VMAT	VMAT	VMAT
1	28.53	17.02	7.96	2.54	0.39	0.00	0.00
2	29.09	6.69	1.07	0.02	0.00	0.00	0.00
3	23.23	11.74	3.48	0.02	0.00	0.00	0.00
4	53.00	35.04	18.45	6.96	0.93	0.00	0.00
5	3.54	0.00	0.00	0.00	0.00	0.00	0.00
6	20.26	3.57	0.50	0.00	0.00	0.00	0.00
7	72.69	23.79	1.91	0.04	0.00	0.00	0.00
8	32.08	7.99	2.06	0.07	0.00	0.00	0.00
9	13.92	5.05	0.99	0.00	0.00	0.00	0.00
10	27.29	14.53	7.95	2.56	0.01	0.00	0.00
11	5.32	0.45	0.00	0.00	0.00	0.00	0.00
12	50.06	15.73	5.47	0.29	0.00	0.00	0.00
13	73.14	27.86	11.20	2.63	0.07	0.00	0.00
14	0.01	0.00	0.00	0.00	0.00	0.00	0.00
15	0.45	0.00	0.00	0.00	0.00	0.00	0.00
16	45.18	6.78	2.20	0.31	0.00	0.00	0.00
17	25.76	5.28	0.04	0.00	0.00	0.00	0.00
18	28.18	21.61	13.72	6.18	1.52	0.00	0.00
19	42.95	12.90	3.87	1.59	0.60	0.21	0.00
20	52.45	14.90	2.08	0.00	0.00	0.00	0.00
21	1.83	0.13	0.00	0.00	0.00	0.00	0.00
22	48.58	6.92	0.97	0.00	0.00	0.00	0.00
23	31.69	13.98	6.00	2.45	0.57	0.00	0.00
24	44.77	33.86	19.81	8.89	2.64	0.19	0.00
25	6.81	0.95	0.08	0.00	0.00	0.00	0.00
26	6.08	2.26	0.13	0.00	0.00	0.00	0.00
27	36.88	20.47	9.10	1.33	0.05	0.00	0.00
28	16.23	2.28	0.03	0.00	0.00	0.00	0.00
29	33.59	8.27	0.26	0.00	0.00	0.00	0.00
30	16.18	5.18	1.11	0.00	0.00	0.00	0.00
31	24.53	6.37	0.98	0.01	0.00	0.00	0.00
32	44.30	17.79	7.54	3.09	0.54	0.00	0.00
33	3.93	0.52	0.00	0.00	0.00	0.00	0.00
34	45.62	25.90	5.47	0.37	0.00	0.00	0.00

#	Femoral head_R (FHR) volume [ccm]	FHR Dmean [Gy]	FHR Dmax [Gy]	FHR V5 [ccm]	FHR V10 [ccm]	FHR V15 [ccm]
		3D-RT	3D-RT	3D-RT	3D-RT	3D-RT
1	131.95	26.79	49.70	112.22	104.11	98.34
2	184.25	28.36	41.01	162.89	152.82	147.63
3	161.80	26.38	45.35	129.91	116.15	108.01
4	160.46	31.24	49.80	152.62	143.46	134.84
5	195.78	27.59	39.37	184.33	175.39	170.41
6	201.96	19.82	43.92	148.27	128.59	118.68
7	134.90	29.01	48.70	123.58	116.48	113.54
8	198.52	31.95	44.69	186.16	172.42	161.39
9	145.68	14.36	49.16	71.26	61.64	56.23
10	154.61	18.84	52.22	99.99	89.29	83.37
11	254.23	25.00	43.47	207.67	185.79	177.86
12	159.97	29.30	46.32	141.75	132.99	129.16
13	251.24	28.10	50.14	203.29	186.08	173.88
14	210.10	22.34	44.93	151.52	133.63	121.85
15	285.00	18.85	46.00	181.95	160.77	152.17
16	123.15	24.14	43.53	97.99	88.86	81.69
17	132.02	20.56	47.13	91.06	80.42	72.88
18	137.29	18.03	48.02	82.66	72.06	64.42
19	132.51	32.40	48.52	130.85	126.03	124.37
20	144.20	28.05	49.91	133.62	121.30	117.81
21	154.75	24.09	45.96	117.97	111.58	109.79
22	115.99	34.29	46.13	112.26	106.14	102.59
23	89.40	23.00	48.69	66.80	59.93	55.13
24	155.52	33.91	48.63	155.52	154.68	152.74
25	175.61	32.74	47.99	167.87	156.01	150.06
26	152.17	15.64	43.70	89.34	76.64	69.70
27	230.06	28.37	49.46	197.90	187.43	183.61
28	214.34	26.45	44.59	188.05	181.43	178.45
29	248.16	33.26	49.70	229.43	216.74	211.90
30	217.23	14.60	40.87	120.53	103.74	93.00
31	134.89	25.41	49.25	107.27	99.86	92.19
32	146.52	30.56	50.78	132.20	123.58	118.22
33	192.88	33.19	41.86	189.38	186.50	184.32
34	129.06	23.66	45.65	93.97	86.62	81.32

#	FHR V20 [ccm]	FHR V25 [ccm]	FHR V30 [ccm]	FHR V35 [ccm]	FHR V40 [ccm]	FHR V45 [ccm]	FHR V50 [ccm]
	3D-RT	3D-RT	3D-RT	3D-RT	3D-RT	3D-RT	3D-RT
1	94.25	87.78	73.45	57.09	6.31	3.04	0.00
2	144.11	136.45	115.57	86.80	6.95	0.00	0.00
3	105.31	103.55	95.17	75.14	34.85	0.63	0.00
4	132.52	128.01	111.51	89.98	24.92	1.79	0.00
5	164.03	138.59	99.76	55.06	0.00	0.00	0.00
6	112.68	85.94	66.42	36.78	3.80	0.00	0.00
7	110.26	101.95	75.43	55.28	15.60	1.05	0.00
8	158.57	155.95	146.67	127.47	53.40	0.00	0.00
9	51.42	46.34	36.16	26.38	4.37	1.69	0.00
10	79.80	68.67	51.31	30.63	5.56	2.22	0.36
11	171.88	150.61	126.46	75.28	7.20	0.00	0.00
12	126.74	117.28	99.65	80.77	25.77	0.06	0.00
13	170.12	166.55	159.51	143.81	75.39	2.47	0.01
14	116.34	106.92	93.03	78.22	36.09	0.00	0.00
15	145.02	128.17	100.33	71.52	2.27	0.02	0.00
16	79.30	73.94	60.50	45.81	3.02	0.00	0.00
17	69.57	66.36	52.44	39.64	6.96	0.39	0.00
18	60.58	56.61	51.21	40.51	3.85	0.22	0.00
19	122.48	119.26	113.17	51.28	3.67	1.03	0.00
20	114.06	107.01	77.85	50.32	12.00	1.63	0.00
21	105.05	95.92	63.18	46.76	17.09	0.02	0.00
22	100.70	99.21	89.95	78.41	47.03	0.03	0.00
23	53.67	50.29	45.49	32.80	2.85	1.12	0.00
24	150.91	146.39	134.05	73.80	3.96	1.56	0.00
25	148.12	141.95	127.52	109.12	59.08	0.29	0.00
26	64.86	53.75	31.94	17.27	3.91	0.00	0.00
27	181.55	173.31	139.99	99.00	22.11	1.78	0.00
28	173.69	140.09	101.91	56.61	7.46	0.00	0.00
29	208.37	206.08	191.67	164.17	91.78	2.03	0.00
30	84.04	65.97	49.45	30.86	0.01	0.00	0.00
31	87.50	81.04	74.31	58.81	11.66	2.08	0.00
32	115.82	112.27	104.59	89.57	13.56	8.85	1.83
33	182.85	177.27	153.12	111.32	1.41	0.00	0.00
34	78.70	75.47	68.77	57.85	4.76	0.02	0.00

#	FHR Dmean [Gy]	FHR Dmax [Gy]	FHR V5 [ccm]	FHR V10 [ccm]	FHR V15 [ccm]
	VMAT	VMAT	VMAT	VMAT	VMAT
1	13.53	43.61	107.82	97.04	46.15
2	10.53	31.45	153.16	85.89	39.36
3	8.04	36.31	92.64	43.00	27.52
4	14.13	42.85	137.34	93.71	56.78
5	9.85	21.36	171.57	82.64	31.90
6	7.97	29.90	122.20	58.49	34.28
7	17.21	39.98	128.26	111.47	91.03
8	11.42	28.07	171.45	120.25	53.09
9	7.21	36.53	63.80	38.43	24.71
10	15.77	42.40	117.56	109.42	88.50
11	9.47	28.29	194.63	105.38	31.14
12	15.83	40.22	143.92	126.20	90.89
13	15.65	43.92	209.96	177.06	143.96
14	4.65	19.71	82.10	15.45	1.06
15	4.77	28.24	90.29	30.44	14.40
16	11.80	37.11	103.91	77.81	36.70
17	10.34	32.19	87.45	70.52	39.62
18	9.55	42.21	68.33	48.71	36.55
19	16.78	35.65	132.51	115.52	71.13
20	17.64	36.20	141.44	117.86	93.46
21	6.65	26.64	101.93	24.50	5.28
22	12.61	36.94	115.51	66.85	29.75
23	11.29	39.58	66.27	50.67	26.50
24	11.95	45.06	146.49	59.74	37.71
25	9.52	28.10	145.73	72.58	22.82
26	6.77	28.80	85.67	34.71	14.56
27	16.46	38.10	193.62	176.12	136.31
28	9.23	25.65	183.75	92.77	14.06
29	10.84	33.29	212.31	118.28	59.43
30	7.56	34.43	123.06	61.32	36.28
31	13.97	42.03	108.11	98.19	58.04
32	16.73	44.76	139.00	124.03	86.92
33	10.84	31.48	184.66	104.67	26.38
34	11.51	32.09	92.46	71.97	41.80

#	FHR V20 [ccm]	FHR V25 [ccm]	FHR V30 [ccm]	FHR V35 [ccm]	FHR V40 [ccm]	FHR V45 [ccm]	FHR V50 [ccm]
	VMAT	VMAT	VMAT	VMAT	VMAT	VMAT	VMAT
1	22.23	11.53	5.32	1.90	0.31	0.00	0.00
2	13.42	41.15	0.03	0.00	0.00	0.00	0.00
3	16.09	6.69	1.27	0.02	0.00	0.00	0.00
4	42.02	24.70	11.40	3.14	0.16	0.00	0.00
5	2.16	0.00	0.00	0.00	0.00	0.00	0.00
6	14.69	1.60	0.00	0.00	0.00	0.00	0.00
7	44.79	17.65	4.34	0.60	0.00	0.00	0.00
8	5.93	0.38	0.00	0.00	0.00	0.00	0.00
9	14.21	7.08	2.13	0.06	0.00	0.00	0.00
10	50.43	26.20	12.67	3.68	0.20	0.00	0.00
11	5.14	0.42	0.00	0.00	0.00	0.00	0.00
12	40.02	17.08	5.90	1.17	0.00	0.00	0.00
13	79.23	32.62	14.39	5.15	0.74	0.00	0.00
14	0.00	0.00	0.00	0.00	0.00	0.00	0.00
15	6.33	1.02	0.00	0.00	0.00	0.00	0.00
16	9.60	1.89	0.58	0.04	0.00	0.00	0.00
17	14.00	1.77	0.14	0.00	0.00	0.00	0.00
18	25.87	14.65	6.23	1.59	0.14	0.00	0.00
19	46.91	9.88	1.40	0.02	0.00	0.00	0.00
20	54.69	24.32	5.42	0.03	0.00	0.00	0.00
21	0.54	0.01	0.00	0.00	0.00	0.00	0.00
22	14.33	5.27	1.43	0.00	0.00	0.00	0.00
23	8.97	4.30	1.60	0.39	0.00	0.00	0.00
24	27.34	16.13	7.07	2.68	0.44	0.00	0.00
25	4.61	0.68	0.00	0.00	0.00	0.00	0.00
26	5.68	0.40	0.00	0.00	0.00	0.00	0.00
27	91.43	37.72	5.28	0.12	0.00	0.00	0.00
28	1.55	0.01	0.00	0.00	0.00	0.00	0.00
29	22.50	3.59	0.29	0.00	0.00	0.00	0.00
30	15.65	5.79	0.76	0.00	0.00	0.00	0.00
31	26.08	11.98	3.53	0.61	0.02	0.00	0.00
32	37.56	18.39	7.16	2.43	0.58	0.00	0.00
33	7.95	1.55	0.03	0.00	0.00	0.00	0.00
34	22.34	5.82	0.75	0.00	0.00	0.00	0.00

Abbreviations

# = patient number, ECOG = Eastern Cooperative Oncology Group, UICC = Union for International Cancer Control, CRM = circumferential resection margin, EMVI = extramural venous invasion, PTV = planning target volume, L4 = lumbar vertebra 4, L5 = lumbar vertebra 5, S1 = sacral vertebra 1, ICT = induction chemotherapy, CRT = chemoradiotherapy, CCT = consolidation chemotherapy, 3D-RT = 3D-conformal radiotherapy, VMAT = volumetric modulated arc therapy, MV = megavolt, ° = grade, ccm = cubic centimeter, BL = bladder, Gy = Gray, BW = bladder wall, SB = small bowel, SP = sphincter, L = left, FHL = femoral head left, R = right, FHR = femoral head right
